# The prognostic significance of [^18^F]FDG PET/CT in multiple myeloma according to novel interpretation criteria (IMPeTUs)

**DOI:** 10.1186/s13550-021-00846-y

**Published:** 2021-10-09

**Authors:** Christos Sachpekidis, Maximilian Merz, Marc-Steffen Raab, Uta Bertsch, Vivienn Weru, Annette Kopp-Schneider, Anna Jauch, Hartmut Goldschmidt, Antonia Dimitrakopoulou-Strauss

**Affiliations:** 1 Clinical Cooperation Unit Nuclear Medicine, German Cancer Research Center (DKFZ), Im Neuenheimer Feld 280, 69210 Heidelberg, Germany; 2 Department of Hematology and Cell Therapy, University of Leipzig, Leipzig, Germany; 3 Department of Internal Medicine V, University Hospital Heidelberg and National Center for Tumor Diseases (NCT), Heidelberg, Germany; 4 Clinical Cooperation Unit Molecular Hematology/Oncology, German Cancer Research Center (DKFZ), Heidelberg, Germany; 5 Division of Biostatistics, German Cancer Research Center (DKFZ), Heidelberg, Germany; 6 Institute of Human Genetics, University of Heidelberg, Heidelberg, Germany

**Keywords:** Multiple myeloma, [^18^F]FDG PET/CT, Italian Myeloma criteria for PET Use (IMPeTUs), Interpretation criteria

## Abstract

**Purpose:**

[^18^F]FDG PET/CT is the elective imaging modality for treatment monitoring in multiple myeloma (MM). However, MM is a heterogeneous disease from an imaging point of view, raising challenges in interpretation of PET/CT. We herein investigated the prognostic role of the novel Italian Myeloma criteria for PET Use (IMPeTUs) in MM patients undergoing high-dose chemotherapy (HDT) followed by autologous stem cell transplantation (ASCT).

**Methods:**

Forty-seven patients with newly diagnosed MM underwent [^18^F]FDG PET/CT before commencement of treatment (baseline PET/CT). Thirty-four of them (72.3%) were also examined after completion of ASCT (follow-up PET/CT). PET/CT analysis was based on the IMPeTUs criteria, which take into consideration—among others—the metabolic state of the bone marrow based on the 5-point Deauville score (DS), the number and metabolic state of focal [^18^F]FDG-avid lesions, as well as the presence of paramedullary disease (PMD) and extramedullary disease (EMD). We analyzed whether parameters from IMPeTUs correlate with clinically relevant parameters and patients’ outcome, as assessed by progression-free survival (PFS).

**Results:**

Median follow-up from baseline and follow-up PET/CT were 85.1 months and 76.7 months, respectively. The number of focal, [^18^F]FDG-avid lesions significantly correlated with the bone marrow infiltration rate and the R-ISS stage, while the presence of PMD was associated with LDH. After univariate survival analysis, the number of focal, [^18^F]FDG-avid lesions both before and after therapy as well as the presence of PMD and EMD before therapy adversely affected PFS. Multivariate survival analysis for baseline parameters confirmed that the number of focal, [^18^F]FDG-avid lesions and the presence of EMD are associated with adverse prognosis, irrespective of the ISS stage and/or the presence of high-risk cytogenetic abnormalities. The 5-point DS of [^18^F]FDG uptake in reference bone marrow and focal lesions showed a significant decrease as response to treatment, but it did not affect PFS.

**Conclusion:**

Several parameters utilized in IMPeTUs predict PFS in MM patients, suggesting the potentially significant role of the new criteria in patient stratification and response assessment. Additional studies are warranted for the further evaluation of IMPeTUs in the direction of establishment of robust cut-off values with a prognostic significance in the disease.

**Supplementary Information:**

The online version contains supplementary material available at 10.1186/s13550-021-00846-y.

## Introduction

[^18^F]FDG PET/CT is considered a valuable tool in the work-up and management of patients with multiple myeloma (MM), due to its ability in detecting both medullary and extramedullary lesions with a high sensitivity and specificity [1–6]. The major strength of the modality is the reliable differentiation between metabolically active and inactive myeloma lesions, rendering it the gold standard for treatment monitoring and response evaluation [3, 4].

On the other hands, several sources of false-positive and false-negative results in PET imaging have been recognized [1, 5, 7]. Moreover, in MM—more than in other diseases—issues on the evaluation of [^18^F]FDG PET/CT scans exist: the different patterns of bone marrow involvement and the non-negligible incidence of concomitant, myeloma-related events such as extramedullary (EMD) and paramedullary disease (PMD) as well as pathological fractures complicate PET/CT image analyses. Additionally, the lower proliferation rate of MM cells leads to a lack of established criteria for PET/CT evaluation and, subsequently, to poor inter-observer reproducibility in interpreting scan results [8–10].

Recently, in an attempt to address the issue of standardization of [^18^F]FDG PET/CT evaluation in MM, a group of Italian experts defined new visual descriptive criteria, the Italian Myeloma criteria for PET Use (IMPeTUs), which take into consideration several parameters of significance in MM (10). Although the initial experience with the IMPeTUs criteria has been promising [11–13], their application as interpretation tool of PET/CT scans is still not broad.

In the current study, we investigate the prognostic significance of the IMPeTUs criteria in MM patients undergoing high-dose chemotherapy (HDT) followed by autologous stem cell transplantation (ASCT).

## Materials and methods

### Patients

Forty-seven consecutive patients (31 male, 16 female; median age 59.9 years) with newly diagnosed, symptomatic MM based on the criteria established by the International Myeloma Working Group (2003) were included in the study [14]. All patients underwent bortezomib-based induction therapies, followed by HDT and ASCT. Twenty-two patients were enrolled in the prospective GMMG MM5 phase III trial that compared two different bortezomib-based induction therapies, followed by ASCT and lenalidomide maintenance therapy for two years or until complete response [15]. Twenty-six patients were treated outside the MM5 trial with comparable treatment regimens and ASCT. This patient cohort has been formerly studied by our group in a different analysis focusing on dynamic [^18^F]FDG PET/CT [16]. In the present study, the patients underwent a longer follow-up period, while the evaluation was based on the application of the IMPeTUs criteria on static whole-body PET/CT images. All patients gave written informed consent after the study was fully explained to them. The study was conducted in accordance with the declaration of Helsinki, with institutional approval by the ethical committee of the University of Heidelberg and the Federal Agency of Radiation Protection in Germany ("Bundesamt für Strahlenschutz").

### PET/CT data acquisition

All patients (*n* = 47) underwent whole-body [^18^F]FDG PET/CT at diagnosis before commencement of treatment (baseline PET/CT). Moreover, 34/47 patients (72.3%) were examined with another PET/CT scan after completion of ASCT and before maintenance therapy (follow-up PET/CT). Thirteen patients of the initial cohort were not included in the second (follow-up) PET/CT analysis due to different reasons (e.g., performance of the second PET/CT already after initiation of maintenance treatment, refusal of patients to undergo another PET/CT scan, or incapacity to perform the second PET/CT at the defined time-point due to practical reasons). Patients were scanned between June 2011 and April 2015. Whole-body PET/CT imaging from the head to the feet was performed 60 min post-injection (p.i.) of [^18^F]FDG. A dedicated PET/CT system (Biograph mCT, S128, Siemens Co., Erlangen, Germany) with an axial field of view of 21.6 cm with TruePoint and TrueV, operated in a three-dimensional mode, was used. A low-dose attenuation CT (120 kV, 30 mA) was used for attenuation correction of the PET data and for image fusion. All PET images were attenuation-corrected, and an image matrix of 400 × 400 pixels was used for iterative image reconstruction. Iterative images reconstruction was based on the ordered subset expectation maximization (OSEM) algorithm with two iterations and 21 subsets as well as time of flight (TOF).

### PET/CT data analysis

[^18^F]FDG PET/CT images were independently analyzed on an Aycan workstation by two nuclear medicine physicians (CS, ADS). Any inter-reader disagreements were resolved by consensus. Image interpretation was based on the IMPeTUs criteria [10], which take into consideration the following parameters:

- Metabolic state of the bone marrow calculated in the lower lumbar spine, if without focal tracer enhancement. This is based on the 5-point Deauville score (DS): score 1, no uptake at all; score 2, ≤ mediastinal blood pool uptake; score 3, > mediastinal blood pool uptake, ≤ liver uptake; score 4, > liver uptake + 10%; score 5, ≫ liver uptake (twice).
- Number of focal, [^18^F]FDG-avid medullary lesions, consistent with MM (Fx): F1, no lesions; F2, 1–3 lesions; F3, 4–10 lesions; F4, > 10 lesions.
- Site of focal, [^18^F]FDG-avid medullary lesions, consistent with MM: skull, spine, other.
- Degree of [^18^F]FDG uptake of the hottest MM lesion, based on DS. In baseline imaging, the focal lesion with the highest metabolic activity was evaluated. In follow-up PET/CT, two approaches were applied: (1) The DS evaluation was performed in the clearly delineated, hypermetabolic focal lesion with the highest uptake in follow-up PET/CT (if present), regardless of the distribution of the hottest lesions in baseline imaging. (2) The DS evaluation was performed in the anatomic area, in which the hottest lesion was detected in baseline PET/CT, irrespective of the presence or absence of a clearly delineated, [^18^F]FDG avid lesion in this localization at follow-up.
- Number of lytic lesions in CT (Lx): L1, no lesions; L2, 1–3 lesions; L3, 4–10 lesions; L4, > 10 lesions.
- The presence of at least one fracture in CT.
- The presence of PMD, defined as a bone lesion extruding through cortical bone and involving surrounding soft tissues.
- The presence of EMD, defined as a myeloma manifestation into soft tissues, with no relationship to the bone.

Semi-quantitative evaluation was based on volumes of interest (VOIs) and on subsequent calculation of standardized uptake value (SUV). VOIs used for SUV calculations of the bone marrow (lower lumbar spine) and the hottest MM lesions were drawn with an isocontour mode [17]. The respective SUV calculations for the reference organs (liver, mediastinum) were performed as suggested by the respective literature [10].

### Bone marrow plasma cell infiltration, clinical parameters, and fluorescence in situ hybridization

Bone marrow biopsies or aspirates from the iliac crest were performed within four weeks around the baseline [^18^F]FDG PET/CT examination and prior to the commencement of treatment in all patients. Percentage of bone marrow infiltration by malignant plasma cells was assessed via light microscope from Giemsa-stained bone marrow smears [18] The infiltration rate represents the number of plasma cell in comparison to all nucleated, hematopoietic cells in the bone marrow. Fluorescence in situ hybridization (FISH) was performed before start of therapy, as described previously [19], on CD138-purified plasma cells using the following probes: 1q21, 5p15, 5q35, 8p21, 9q34, 11q22.3, 13q14, 15q22, 17p13, and 19q13. We also investigated immunoglobulin H (IgH) translocations using an IgH break-apart probe as well as probes for t(11;14), t(4;14), and t(14;16). Patients were staged according to the MM International Staging System (ISS) [20]. Moreover, the Revised International Staging System (R-ISS) score was defined for the definition of high-risk disease [21].

### Statistical analysis

Depending on the variables tested, the following approaches were applied for correlation and association analysis: for the correlation between continuous variables Spearman rank correlation coefficient, between nominal and ordinal variables chi-square tests of association, between continuous and nominal variables Kruskal–Wallis test, and between continuous and ordinal variables Jonckheere–Terpstra test. Concerning association between nominal variables, chi-square tests of association were computed: For nominal variables with only two levels each, phi coefficient was calculated, while for those with more than two levels, Cramer’s v was computed. Comparisons between baseline and follow-up parameters were performed with use of paired t-test for continuous variables, McNemar test for nominal variables, and Wilcoxon signed-rank test for the “score” variables (DS of the bone marrow, DS of the hottest lesion, number of focal lesions-Fx, number of lytic lesions-Lx). Progression-free survival (PFS) was measured from the date of baseline (*n* = 47 patients) and follow-up (*n* = 34 patients) PET/CT, respectively, until disease progression or death from any cause. Kaplan–Meier estimates were generated and median PFS estimated. Median follow-up time was determined by inverse Kaplan–Meier estimation. For univariate comparison of PFS log-rank test was used. Univariable Cox proportional hazards regression analysis was applied for the continuous variables. Multivariate Cox proportional hazards regression analysis was also applied. Statistical analysis was performed using R version 3.4.3 (The R Foundation for Statistical Computing 2017) and R packages survival, survminer and prodlim. The results were considered significant for p value less than 0.05 (*p* < 0.05). The reported analyses are exploratory, and no correction for multiplicity of testing was made.

## Results

### Patient characteristics

According to ISS, 26 patients were classified in stage I (61.9%), 12 patients in stage II (28.6%), and four patients in stage III (9.5%). The median bone marrow plasma cell infiltration rate before commencement of treatment was 32% (range 1–92%). Cytogenetic data were available in 40 patients of the studied cohort (80%), with high-risk abnormalities being detected in eight of them (20%). A combination of the ISS and cytogenetic data was available in 36 patients, leading to the following classification according to R-ISS: 14 patients in group R-ISS-I (38.9%), 20 patients in group R-ISS-II (55.5%), and two patients in group R-ISS-III (5.6%). The characteristics of the studied patients at baseline are presented in Table 1.

**Table 1:** Baseline patient characteristics

Baseline characteristics	n (%) [range]
Median age, years (47 patients)	59.9 [38.4–73.5]
Gender (47 patients)	
Male	31 (66%)
Female	16 (34%)
Median albumin, g/dl (44 patients)	4.2 [0.5–5.0]
Median β2-microglobulin, mg/l (43 patients)	2.8 [1.1–37.0]
Median LDH, u/l (45 patients)	184.0 [117.0–283.0]
Median bone marrow plasma cell infiltration (45 patients)	32% [1%–92%]
Cytogenetic abnormalities (40 patients)	
High-risk	8 (20.0%)
Standard risk	32 (80.0%)
ISS (42 patients)	
I	26 (61.9%)
II	12 (28.6%)
III	4 (9.5%)
R-ISS (36 patients)	
I	14 (38.9%)
II	20 (55.6%)
III	2 (5.6%)

### [^18^F]FDG PET /CT findings

#### Baseline PET/CT

The application of IMPeTUs revealed the following results: the median five-point DS of diffuse bone marrow uptake was 3 (range DS = 2–5). Eleven patients (23%) had no visually detectable focal, medullary, hypermetabolic lesions (F1 score), while 36 of them (77%) had at least one focal hypermetabolic lesion (median F score = 2; range F score = 1–4). In the 36 patients with focal lesions, the median five-point DS of the hottest lesion was 5 (range DS = 3–5). PMD and EMD were present in 23/47 (49%) and 4/47 patients (9%), respectively. Regarding EMD, its site was nodal in two patients and extranodal (intramuscular) in the other two. Eight patients (17%) had no osteolysis (L1 score), while 39 of them (83%) showed at least one lytic lesion (median L score = 3; range L score = 1–4). Fractures were detected in 22 patients (47%).

With concern to inter-reader agreement in application of IMPeTUs, there were three cases (6%) of discordant findings regarding the number of [^18^F]FDG-avid medullary lesions (Fx) in baseline PET/CT. Respectively, in four patients (9%), there was disagreement regarding the number of lytic lesions in CT (Lx). On the other hands, no disagreement occurred with regard to the DS-based classification of patients in groups according to the degree of [^18^F]FDG uptake in the bone marrow and the hottest focal lesions. Further, there were no discordant findings regarding the presence of fractures, PMD, or EMD in the studied patients. All cases of inter-reader disagreement were resolved by consensus.

#### Follow-up PET/CT

**Fig. 1:**
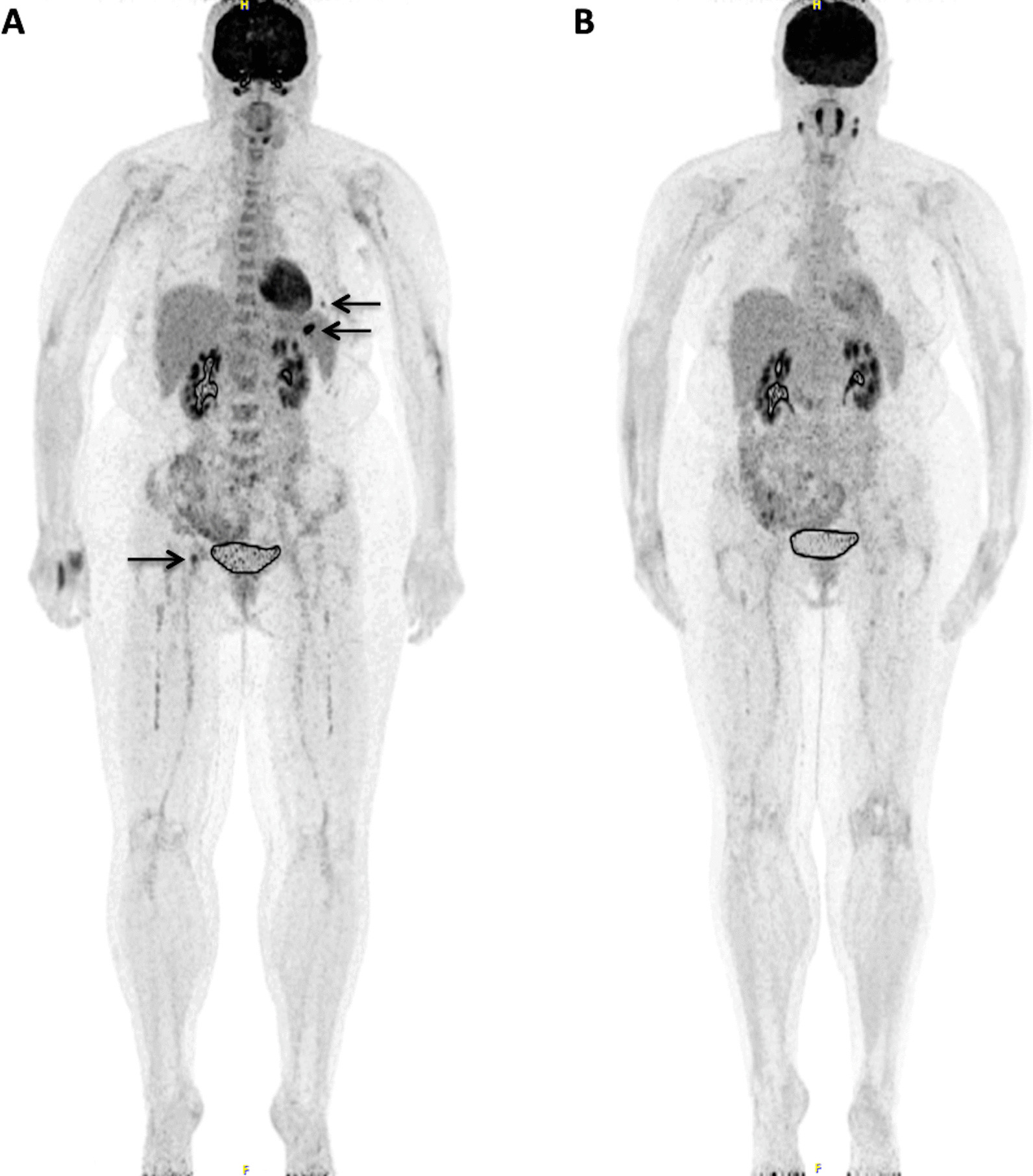
A 59-year-old female MM patient undergoing [^18^F]FDG PET/CT before and after HDT and ASCT. **A** Maximum intensity projection (MIP) PET/CT before therapy revealed an increased, diffuse bone marrow uptake of the tracer in the axial skeleton and the proximal parts of both humeri and femora, as well as several focal bone marrow lesions (arrows). According to IMPeTUs, the patient was classified at baseline as follows: BM4, F3 Sp, ExtraSp (DS5), L2. **B** Follow-up [^18^F]FDG PET/CT after ASCT demonstrated a complete remission of both diffuse bone marrow uptake as well as focal medullary lesions. [^18^F]FDG uptake in cervical lymph nodes was attributed to inflammatory reaction after therapy, thus considered benign. The respective results according to IMPeTUs at follow-up were: BM3, F1, L2, Fr

**Fig. 2:**
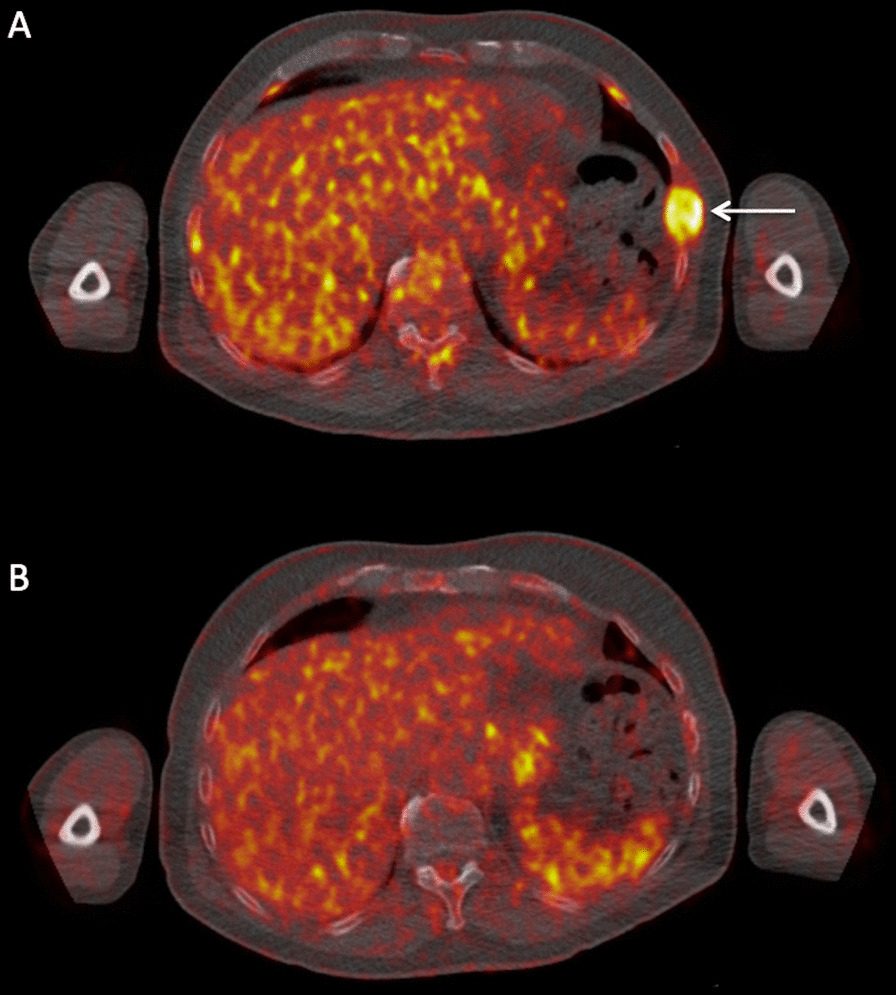
Transaxial PET/CT images at the thoracic level of a 70-year-old, male MM patient before and after therapy. **A** Baseline PET/CT revealed a hypermetabolic, focal lesion in the 8th left rib with cortical bone interruption and involvement of the surrounding soft tissues (arrow) as a sign of PMD. **B** After completion of HDT and ASCT, follow-up PET/CT revealed a complete metabolic remission of the former hypermetabolic PMD and development of a rib fracture

**Fig. 3:**
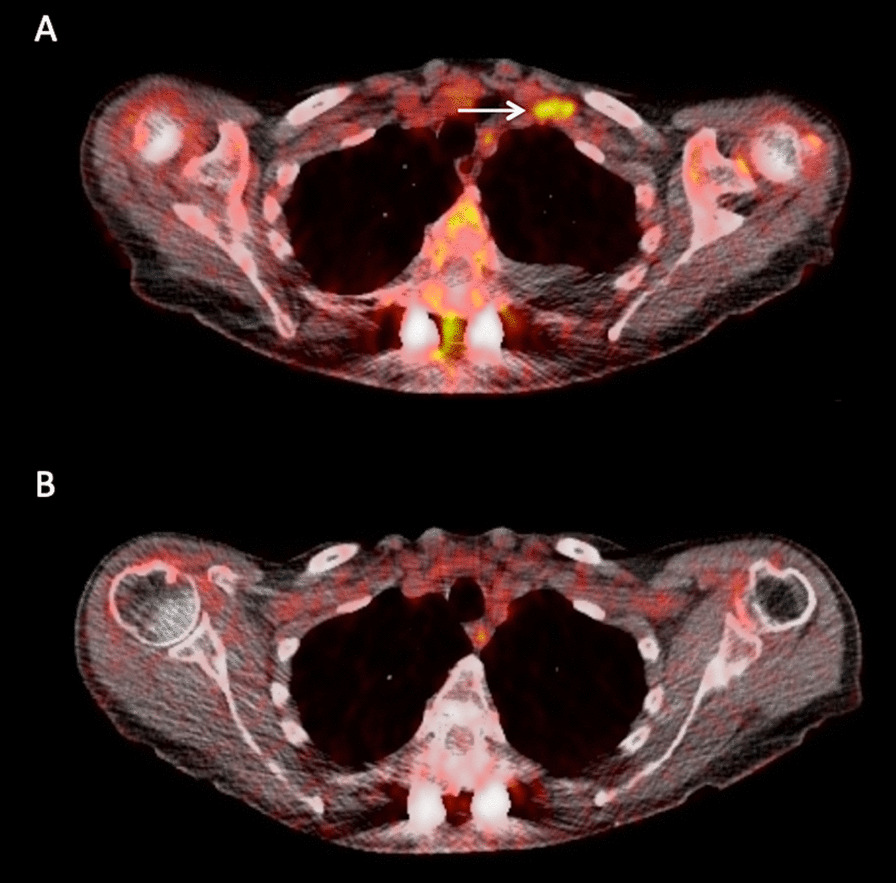
Transaxial PET/CT images at the thoracic level of a 69-year-old female MM patient before and after therapy. **A** Baseline PET/CT demonstrated a hypermetabolic, intramuscular, soft-tissue lesion in the thoracic wall with no relationship to the bone (arrow) as a sign of EMD. **B** Follow-up PET/CT showed a complete metabolic remission of the EMD as response to treatment (**B**)

After ASCT, a significant decrease in diffuse bone marrow uptake was observed compared to baseline (*p* < 0.001). In particular, at follow-up 32/34 (94%), patients had DS 2 or 3, and only two patients DS 4 or 5 (6%) (median DS = 3; range DS = 2–4). Similarly, at follow-up the number of focal, medullary, hypermetabolic lesions (Fx) decreased significantly from baseline (*p* < 0.001): 21/34 patients (62%) showed no lesions (F1 score), while 13/34 patients (38%) showed at least one lesion (median F score = 1; range F score = 1–4) (Fig. 1). With regard to the 13 patients with clearly delineated, hypermetabolic focal lesions at follow-up, the median DS of the hottest lesions was 4 (range DS = 2–5), not exhibiting a significant difference with baseline (*p* = 0.188); these DS estimations refer to the lesions clearly demonstrated in follow-up PET/CT, irrespective of the distribution of lesions in baseline imaging (Approach 1). On the other hands, according to Approach 2, a significant DS decrease was observed between pre- and post-treatment PET/CT in the anatomic areas, in which the hottest lesions were detected in baseline PET/CT (median DS = 3; range DS = 1–5) (*p* < 0.001). A significant decrease was observed in the incidence of hypermetabolic PMD, with no patient showing such signs in follow-up PET/CT (*p* < 0.001) (Fig. 2). In contrary, the incidence of EMD did not significantly change from baseline imaging (*p* = 0.414) (*n* = 4/34 patients; 12% at follow-up): Although all baseline manifestations of EMD receded after treatment (Fig. 3), three patients demonstrated new signs of nodal EMD and one patient signs of extranodal (pulmonary) EMD at follow-up. Finally, no significant differences in the number of lytic lesions (Lx) were observed (*p* = 0.625) (median L score = 3; range L score = 1–4), while the incidence of fractures significantly increased (*p* = 0.02) (*n* = 24/34 patients, 71%).

Regarding inter-reader agreement, three cases (9%) of discordant findings in estimation of the number of focal hypermetabolic lesions (Fx) and lytic lesions (Lx) were noted in follow-up PET/CT. Similarly to baseline imaging, no disagreement occurred with regard to the DS-based degree of [^18^F]FDG uptake in the bone marrow and the hottest focal lesions as well as the presence of fractures, PMD, and EMD.

The PET/CT results after application of IMPeTUs are summarized in Tables 2 and 3. Moreover, the SUV values derived from the bone marrow (lower lumbar spine), the hottest MM lesions, the liver, and the mediastinum are presented in Additional file 1.

**Table 2:** PET/CT results after application of IMPeTUs

IMPeTUs criteria	Baseline PET/CT (*n* = 47) Patients (%)^§^	Follow-up PET/CT (*n* = 34) Patients (%)^§^	
*Bone marrow uptake, DS*			
1	0	0	
2	7 (15%)	13 (38%)	
3	19 (40%)	19 (56%)	
4	18 (38%)	2 (6%)	
5	3 (6%)	0	
*No. of focal, hypermetabolic lesions*			
F_1_ (none)	11 (23%)	21 (62%)	
F_2_ (1–3)	15 (32%)	8 (24%)	
F_3_ (4–10)	9 (19%)	3 (9%)	
F_4_ (> 10)	12 (26%)	2 (6%)	
Site of focal lesions*			
Skull	2 (4%)	1 (3%)	
Spine	23 (49%)	3 (9%)	
Other	33 (70%)	12 (35%)	
*Uptake of the hottest focal lesion, DS*		*Approach 1*	*Approach 2*
1	0	0	2 (8%)
2	0	1 (8%)	9 (35%)
3	1 (3%)	0	5 (19%)
4	14 (39%)	7 (54%)	7 (27%)
5	21 (58%)	5 (39%)	3 (12%)
*No. of lytic lesions*			
L_1_ (none)	8 (17%)	5 (15%)	
L_2_ (1–3)	12 (26%)	9 (27%)	
L_3_ (4–10)	7 (15%)	5 (15%)	
L_4_ (> 10)	20 (43%)	15 (44%)	
*Presence of at least one fracture*			
No	25 (53%)	10 (29%)	
Yes	22 (47%)	24 (71%)	
Presence of PMD*			
No	24 (51%)	34 (100%)	
Yes	23 (49%)	0	
Presence of EMD*			
No	43 (92%)	30 (88%)	
Yes	4 (9%)	4 (12%)	

^*^Measurements refer to hypermetabolic lesions

^§^Due to rounding, the % percentage values do not necessarily add to 100%

**Table 3:** Descriptive statistics including median values and range of PET/CT variables after application of IMPeTUs at baseline (before treatment) and follow-up (after ASCT and before maintenance) scanning

	Bone marrow uptake, DS	No. of focal, hypermetabolic lesions	Uptake of the hottest focal lesion, DS		No. of lytic lesions
*Baseline PET/CT*					
Median	3	2	5	3	
Range	2 – 5	1–4	3–5	1–4	
*Follow-up PET/CT*					
Median	3	1	4 *	3 ^§^	3
Range	2 – 4	1–4	2–5 *	1–5 ^§^	1–4
*p* value	< 0.001	< 0.001	0.188^*^	< 0.001^§^	0.625

^*^ Evaluations based on Approach 1

^§^ Evaluations based on Approach 2

### Correlation between baseline [^18^F]FDG PET/CT findings and clinical parameters

Exploratory correlation analysis revealed that the number of focal, [^18^F]FDG-avid medullary lesions (Fx) correlated with the bone marrow infiltration rate (*p* = 0.032) and had an association with R-ISS stage (*p* = 0.016). No significant association was observed regarding the relationship between bone marrow infiltration rate and the score of focal lesions (Fx) after taking into account the different anatomical locations (site) of the lesions (Additional file 2). Moreover, patients with PMD had significantly higher LDH plasma levels than those without PMD (*p* = 0.015).

### Survival analysis

#### Baseline PET/CT

**Fig. 4:**
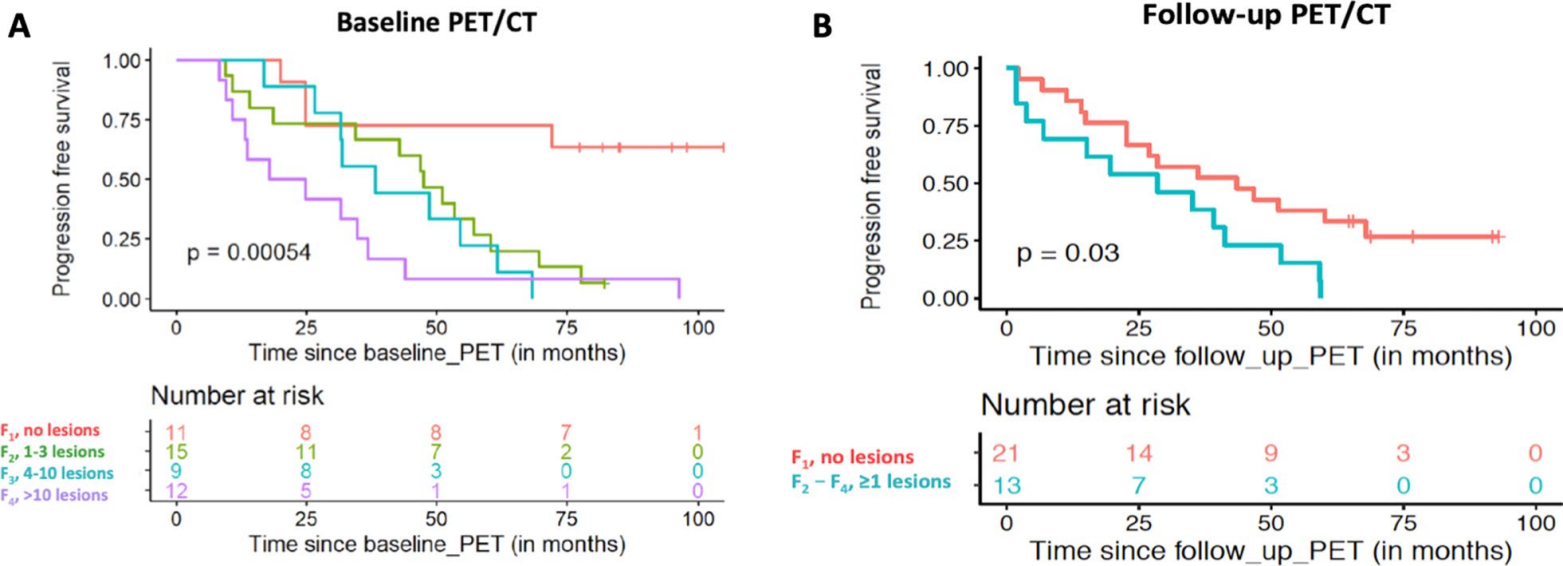
Kaplan–Meier estimates of PFS according to the number of focal, hypermetabolic lesions (Fx) at baseline (**A**) and follow-up PET/CT (**B**). At baseline, the patients are divided according to the four groups of the Fx score (**A**), while at follow-up, they are dichotomized in those having no lesions (F1), and those presenting with at least one lesion (F2–F4). The numbers of patients at risk in each group and for the respective time-points are shown below the plots

**Fig. 5:**
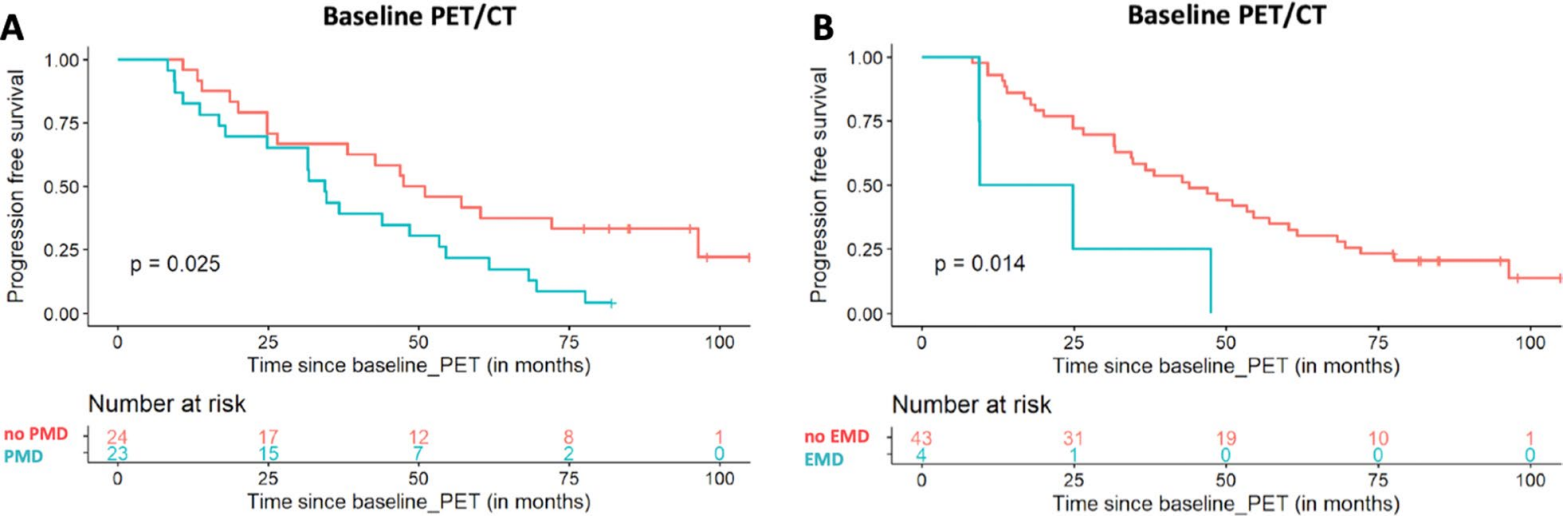
Kaplan–Meier estimates of PFS according to the presence of PMD (**A**) and EMD (**B**) at baseline PET/CT. The numbers of patients at risk in each group and for the respective time-points are shown below the plots

Median follow-up [95% CI] from baseline PET/CT was 85.1 months [82.1–97.9 months]. During this follow-up period, 38 patients had shown disease progression and 9 patients had no progression. Thirty-two patients were still alive, while 15 of them had died. Univariate analysis revealed that the following IMPeTUs parameters derived from baseline PET/CT were associated with a worse PFS. In particular, the number of focal hypermetabolic lesions (Fx) adversely affected survival, with an increasing number of lesions conferring worse PFS (*p* < 0.001) (Fig. 4). Further, patients with PMD had a median PFS of 34.5 months [24.8–54.5] compared to a median PFS of 49.3 months [38.2–NA] for patients without PMD (*p* = 0.025) (Fig. 5). EMD-positive patients had a median PFS of 17.2 months [9.4–NA], while EMD-negative patients had a median PFS of 43.9 months [31.8–61.7] (*p* = 0.014) (Fig. 5). In contrast, the metabolic state of the bone marrow in the lower lumbar spine dichotomized at the median DS value of 3 did not significantly affect survival (*p* = 0.47). Concerning the parameter DS of the hottest MM lesion, the number of patients with DS ≤ 3 was very small to perform survival analysis based on this cut-off value (*n* = 1 patient); instead, we used DS 4 as cut-off, which did not have, however, an effect on PFS (*p* = 0.81). Finally, neither the number of osteolysis (Lx) (*p* = 0.1) nor the presence of fractures (*p* = 0.24) in CT was associated with a worse PFS (Table 4).

**Table 4:** Predictors of PFS by univariate analysis based on PET/CT results after application of IMPeTUs

IMPeTUs criteria	PFS	
	Median [95% CI]	
	Baseline PET/CT	Follow-up PET/CT
*Bone marrow uptake, DS score*		
≤ 3	47.2 [31.8–69.6]	31.8 [22.6–51.8]
> 3	34.7 [17.9–72.1]	41.2 [41.2–NA]
*No. of focal, hypermetabolic lesions*		
F_1_ (none)	NA [72.1–NA]*	43.5 [22.6–NA]**
F_2_ (1–3)	47.5 [34.5–69.6]*	37.1 [28.5–NA]**
F_3_ (4–10)	38.2 [31.6–NA]*	15.2 [1.8–NA]**
F_4_ (> 10)	21.4 [13.3–NA]*	13.3 [7.0–NA]**
*Uptake of the hottest focal lesion, DS*		
≤ 3	NA	31.7 [22.6–51.8]^*¶*^
> *3*	NA	24.0 [7.0–NA]^*¶*^
*No. of lytic lesions*		
L_1_ (none)	42.8 [24.8–NA]	NA [36.1–NA]
L_2_ (1–3)	54.1 [36.8–NA]	46.7 [28.4–NA]
L_3_ (4–10)	24.8 [20.1–NA]	11.3 [1.8–NA]
L_4_ (> 10)	39.3 [31.6–61.7]	26.9 [19.6–59.1]
*Presence of at least one fracture*		
No	47.5 [31.8–72.1]	41.4 [26.9–NA]
Yes	35.7 [24.8–61.1]	28.5 [19.6–59.1]
*Presence of PMD*		
No	49.3 [38.2–NA]^#^	35.6 [22.6–51.8]
Yes	34.5 [24.8–54.5]^#^	NA°
*Presence of EMD*		
No	43.9 [31.8–61.7]^§^	37.6 [22.6–59.3]
Yes	17.2 [9.4–NA]^§^	24.8 [2.2–NA]

NA, not applicable

NA°, not applicable, since no patient showed PMD on follow-up PET/CT

*^,#,§^*p* < 0.05, after usage of log-rank test for univariate comparison of PFS

**p < 0.05, after usage of log-rank test for univariate comparison of PFS, based on the dichotomization presence (F_1_) *vs.* absence (> F_1_) of focal lesions on follow-up PET/CT

^¶^The herein presented evaluations in follow-up PET/CT were performed based on Approach 2. According to Approach 1, the number of patients with DS ≤ 3 is too small (*n* = 1 patient) to perform survival analysis based on this cut-off value

Multivariate Cox regression analysis of baseline parameters accounting for R-ISS stage showed that the number of focal, [^18^F]FDG-avid, medullary lesions and the presence of EMD are associated with significantly shorter PFS, irrespective of the ISS stage and/or the presence of high-risk chromosomal abnormalities.

#### Follow-up PET/CT

Median follow-up [95% CI] from the second PET/CT was 76.7 months [65.4 – 91.7 months]. The only IMPeTUs parameter from follow-up PET/CT conferring a worse survival was the number of focal hypermetabolic lesions dichotomized at F1: patients showing at least one focal, [^18^F]FDG avid lesion (> F1) had a median PFS of 28.5 months [7.0–NA], compared to a median PFS of 43.5 months [22.6–NA] for patients without any lesions (F1) (*p* = 0.03) (Fig. 4). The rest of the parameters, including the metabolic state of the bone marrow dichotomized at the median DS 3 (*p* = 0.3), the metabolic state of the hottest lesion dichotomized at the median DS 4 for Approach 1 (*p* = 0.21), and at the median DS 3 for Approach 2 (*p* = 0.52), the presence of EMD (*p* = 0.14), the number of osteolysis (Lx) (*p* = 0.08) and the presence of fractures (*p* = 0.63), did not significantly affect PFS (Table 4).

## Discussion

The recent development of the IMPeTUs criteria aimed to serve the cause of PET/CT analysis in a standardized and simple fashion, based exclusively on visual interpretation of the scans. The preliminary results of the application of these criteria have been promising, yielding a high reproducibility in scan interpretation [10, 11]. In the present study, we investigated the prognostic significance of IMPeTUs in MM patients before and after treatment. The major findings from our analysis are the following: First, upon univariate analysis, the number of focal, medullary, hypermetabolic lesions on PET/CT (Fx) both before and after therapy, as well as the presence of PMD and EMD, have an adverse prognostic significance in patient survival. Second, multivariate survival analysis for baseline parameters confirms that the number of focal, [^18^F]FDG-avid, medullary lesions, and the presence of EMD are associated with adverse prognosis, irrespective of the ISS stage and/or the presence of high-risk chromosomal abnormalities. Third, the 5-point DS of [^18^F]FDG uptake in reference bone marrow and focal lesions significantly decreases as response to treatment, but it does not affect survival.

The number of focal [^18^F]FDG-avid lesions (Fx) demonstrated a prognostic significance both before and after therapy. Specifically, on baseline PET/CT, a higher Fx score was associated with a shorter PFS. This finding was also confirmed via multivariate analysis of baseline parameters accounting for ISS stage and/or the presence of high-risk cytogenetic abnormalities. Respectively, on follow-up PET/CT, the presence of at least one focal lesion (> F1) conferred a worse PFS compared to the absence of any lesions (F1). These findings highlight the clinical relevance of the cut-off values chosen to classify patients in groups based on the number of focal lesions, and they, moreover, confirm the association between the persistence of PET-positive lesions after treatment and impaired clinical outcome [22–24]. Importantly, the number of focal lesions at baseline also correlated significantly with two other clinically relevant parameters in MM, namely the bone marrow infiltration rate and the R-ISS stage.

Another result of our analysis was the adverse effect on survival of the presence of hypermetabolic, soft-tissue components (EMD, PMD). EMD is a recognized prognostic factor for worse survival in MM [23, 25, 26] and the herein presented results confirm this in the pre-treatment setting through both univariate and multivariate survival analysis. Interestingly, however, the presence of EMD in follow-up PET/CT did not confer a worse PFS. A potential explanation for this lies in the assumption that they do not represent true EMD but rather post-therapeutic, hypermetabolic, inflammatory reactions to treatment. This argument is supported, firstly, by the distribution of the PET findings—including an almost symmetric, mediastinal/hilar lymphadenopathy (*n* = 2 patients), a generalized, bilateral lymphadenopathy involving mediastinal, iliac, and inguinal lymph nodes (*n* = 1 patient), and a lung infiltration pattern similar to pneumonitis (*n* = 1 patient) [27, 28]—and, secondly, by the patients’ clinical response after ASCT according to the clinical gold standard (complete response, 2 patients; near-complete response, 1 patient; partial response, 1 patient) [29, 30]. Although not available, a histopathological validation of the imaging findings could definitively rule out false-positive findings. Therefore, in cases of discordancy between imaging and clinical/laboratory findings, especially in the context of measurable residual disease, a histopathological clarification of the equivocal findings is strongly recommended.

On the other hands, the role of PMD in PET/CT has not been investigated as thoroughly as that of EMD. Rasche et al. have shown in a cohort of 404 newly diagnosed MM patients that PMD was present in 36.9% of them, and it, moreover, conferred a shorter PFS [31]. We herein confirm that the presence of hypermetabolic PMD at baseline is not uncommon, affecting 49% of the cohort, it confers an adverse PFS—at least at univariate analysis, and is associated with significantly higher LDH plasma levels, a parameter with major impact on the survival of myeloma patients [32].

An important aspect of IMPeTUs is the introduction of the 5-point DS, originally applied in Hodgkin lymphoma [33, 34], in the assessment of myeloma skeletal involvement. The application of the Deauville criteria before treatment in reference bone marrow and focal myeloma lesions did not reveal a significant effect on PFS. These findings are consistent with the results of a retrospective evaluation of IMPeTUs in 47 MM patients before commencement of treatment, in which the application of the 5-point DS was also not predictive of progression or death. At odds with our findings, however, the authors of that study reported no effect of the number of focal lesions or the presence of soft-tissue components on survival [35].

At follow-up, the DS of reference bone marrow significantly decreased as response to treatment. With respect to focal lesions, two approaches were followed in their assessment: the first approach involved DS estimation of the clearly delineated, focal lesion with the highest uptake in follow-up PET/CT, irrespective of the localization of the hottest lesion in baseline imaging. This approach revealed a non-significant decrease in DS as response to treatment. In the second approach, the DS evaluation in follow-up PET/CT involved the particular anatomic area, in which the hottest lesion was detected in baseline PET/CT, irrespective of the presence or absence of a clearly delineated, [^18^F]FDG-avid lesion in this localization after therapy. Based on this method, a significant decrease of DS was observed after treatment. Similarly, to baseline PET/CT, survival analysis revealed no significant effect of follow-up DS, derived both from reference bone marrow and focal lesions, on PFS.

Recently, the results of a large joint analysis (*n* = 228 patients) involving PET/CT evaluations based on the Deauville criteria were published [13]. Similar to our results, a marked decrease from baseline to follow-up imaging was observed in the incidence of patients with focal lesions as well as in the DS of reference bone marrow. On the other hands, a significant association was reported between post-treatment DS < 4 of both reference bone marrow and focal lesions with prolonged survival. These results are seemingly inconsistent with our respective findings. However, the explanation for this discordance may lie in the markedly lower number of patients enrolled in our study. In specific, at follow-up imaging, only 2/34 patients of our cohort had DS ≥ 4 of the reference bone marrow, compared to 17/195 patients in the aforementioned study. Respectively, at follow-up imaging, only 1/13 patient had DS < 4 of focal lesions—compared to 20/62 patients—according to Approach 1, which is also the method applied in the above-mentioned study [13]. Therefore, the extraction of robust conclusions on patient survival based on these DS cut-off values is not yet feasible by our analysis.

We note some limitations in our study. Foremost, this is a retrospective analysis of prospectively acquired data from a relatively small patient cohort, not allowing the drawing of more firm conclusions, particularly with regard to the predictive role of the 5-point DS on patient survival; further studies with larger patient cohorts would be warranted to validate the here presented findings. Further, the vast majority of the PET/CT-positive, myeloma-consistent findings were not histopathologically confirmed, which is usually not done in the clinical setting. However, we are already in the process of evaluating the performance of image-guided biopsies to molecularly characterize PET-positive lesions [36].

## Conclusion

In an attempt to generate further knowledge on the issue of standardization of [^18^F]FDG PET/CT interpretation in MM, we investigated the prognostic significance of the newly introduced, visual IMPeTUs criteria in MM patients undergoing HDT followed by ASCT. Our results showed via univariate analysis that several parameters utilized in IMPeTUs, including the number of focal, [^18^F]FDG-avid lesions before and after treatment, as well as the presence of PMD and EMD before treatment, significantly affect patient survival. Moreover, multivariate survival analysis for baseline parameters confirmed that the number of focal, [^18^F]FDG-avid lesions and the presence of EMD are associated with adverse prognosis, irrespective of the ISS stage and/or the presence of high-risk chromosomal abnormalities. With regard to the 5-point DS of [^18^F]FDG uptake, originally applied in Hodgkin lymphoma, it showed a significant decrease as response to treatment both in reference bone marrow and focal lesions, but it did not affect patient survival. Although further studies in larger cohorts are necessary for validation, our findings highlight the potentially significant role of the new criteria in myeloma patient stratification and response assessment.

## Supplementary Information

**Additional file 1.** Median SUV values (range) derived from the bone marrow, the hottest MM lesions, the liver and the mediastinum at baseline (before treatment) and follow-up (after ASCT and before maintenance) PET/CT.

**Additional file 2.** Analysis on the relationship between bone marrow plasma cell infiltration percentage and the IMPeTUs parameter number of focal lesions (Fx) after taking into account the different anatomical locations of the lesions.

## Data Availability

The dataset used and/or analyzed during the current study is available from the corresponding authors on reasonable request.
