# Supplementary material for: The prognostic significance of [18F]FDG PET/CT in multiple myeloma according to novel interpretation criteria (IMPeTUs)
Source: EJNMMI Res. 2021 Oct 9;11:100. doi: 10.1186/s13550-021-00846-y (PMC8502185; doi:10.1186/s13550-021-00846-y)
Supplement: Supplementary file 1 — Additional file 1. Median SUV values (range) derived from the bone marrow, the hottest MM lesions, the liver and the mediastinum at baseline (before treatment) and follow-up (after ASCT and before maintenance) PET/CT. [file 13550_2021_846_MOESM1_ESM.docx]

**Supplementary File (Table) 1** Median SUV values (range) derived from the bone marrow, the hottest MM lesions, the liver and the mediastinum at baseline (before treatment) and follow-up (after ASCT and before maintenance) PET/CT.

|  | **Bone marrow**  **(lower lumbar spine)** | | **Hottest MM lesion** | | **Liver** | | **Mediastinum** | |
| --- | --- | --- | --- | --- | --- | --- | --- | --- |
|  | **SUV_mean_** | **SUV_max_** | **SUV_mean_** | **SUV_max_** | **SUV_mean_** | **SUV_max_** | **SUV_mean_** | **SUV_max_** |
| **Baseline PET/CT** | 2.6 (0.9 – 5.6) | 4.2 (1.6 – 11.7) | 5.1 (3.2 – 21.0) | 8.4 (3.7 – 36.0) | 2.6 (1.4 – 4.8) | 4.0 (2.0 – 6.8) | 2.3 (1.7 – 6.2) | 2.8 (2.0 – 6.7) |
| **Follow-up PET/CT** | 1.7 (1.0 – 3.0) | 2.6 (1.6 – 6.3) | 4.1 (1.9 – 6.5) | 6.0 (2.5 – 10.2) | 2.0 (1.5 – 3.3) | 3.2 (2.5 – 5.5) | 1.7 (1.0 – 2.6) | 2.3 (1.7 – 3.7) |
| ***p* value** | <0.001 | <0.001 | 0.019 | 0.004 | <0.001 | 0.006 | <0.001 | 0.003 |
