# Supplementary material for: The prognostic significance of [18F]FDG PET/CT in multiple myeloma according to novel interpretation criteria (IMPeTUs)
Source: EJNMMI Res. 2021 Oct 9;11:100. doi: 10.1186/s13550-021-00846-y (PMC8502185; doi:10.1186/s13550-021-00846-y)
Supplement: Supplementary file 2 — Additional file 2. Analysis on the relationship between bone marrow plasma cell infiltration percentage and the IMPeTUs parameter number of focal lesions (Fx) after taking into account the different anatomical locations of the lesions. [file 13550_2021_846_MOESM2_ESM.docx]

**Supplementary File 2** Analysis on the relationship between bone marrow plasma cell infiltration percentage and the IMPeTUs parameter number of focal lesions (Fx) after taking into account the different anatomical locations of the lesions.

According to IMPeTUs, the site of focal lesions is classified in three groups: skull, spine, and other (all the rest localizations). In total, in the herein analyzed cohort four different combinations of lesions’ localization were documented: A. spine and all the rest,

B. all the rest,

C. skull, spine and all the rest, and

D. skull

The respective plots describing the relationship between bone marrow plasma cell infiltration percentage (y-axis) and the number of focal lesions (Fx) are presented below (Figure 1). Notably, the bone marrow plasma cell infiltration rate has been log-transformed for better assessment. Moreover, the Fx score is an ordered categorical variable, with the following possible values: F1, no lesions; F2, 1 - 3 lesions; F3, 4 - 10 lesions; F4, > 10 lesions.


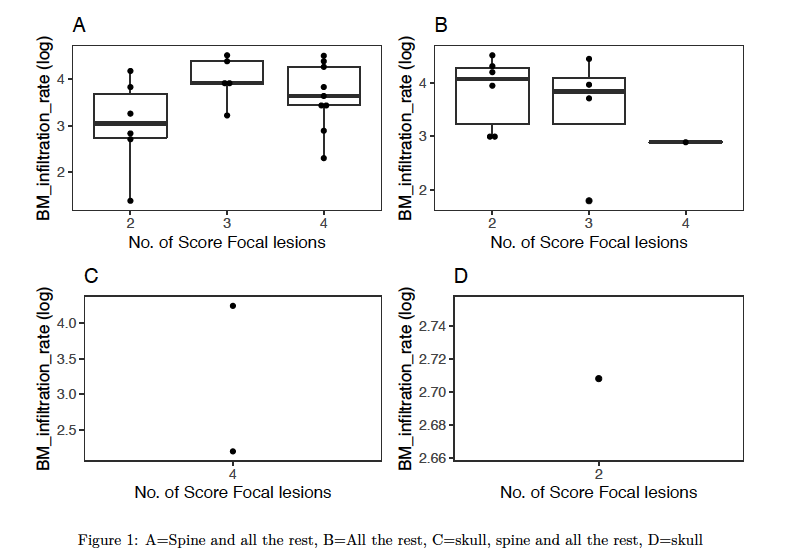


Further, quantitative correlation analysis was performed between the variable number of focal lesions (Fx) and bone marrow plasma cell infiltration percentage regarding the location of the lesions. In particular, the Jonckheere-Terpstra test was used to evaluate whether there is a statistically significant trend (monotone relationship) regarding the relationship between bone marrow infiltration rate and the score of focal lesions (Fx) after taking into account the different anatomical locations. The results of this analysis revealed no significant associations. These results are presented below in detail:

- For focal lesions located in the “spine and all the rest” category (A), there was no statistically significant association between the number of focal lesions (Fx) and BM infiltration rate (p-value=0.3437).
- For focal lesions located in the “all the rest” category (B), there was also no statistically significant association between the number of focal lesions (Fx) and BM infiltration rate (p-value=0.2888).
- For focal lesions located in the “skull, spine and all the rest” category (C), this association could not be evaluated due to limited data.
- For focal lesions located in the “skull” category (D), this association could not be evaluated due to limited data.
